# TSA-Net: Multivariate Time Series Anomaly Detection Based on Two-Stage Temporal Attention

**DOI:** 10.3390/s26031062

**Published:** 2026-02-06

**Authors:** Hao Wu, Wu Le, Zhen-Hong Jia, Hui Zhao, Sai Zhang, Zhen-Sen Zhang

**Affiliations:** 1 Xinjiang Space-Air-Ground Integrated Intelligent Computing Technology Laboratory, Changji 831100, China; 2 School of Computer Science and Technology, Xinjiang University, Urumqi 830046, China; 3 Xinjiang Uygur Autonomous Region Signal Detection and Processing Key Laboratory, Urumqi 830046, China

**Keywords:** anomaly detection, spatio-temporal attention, graph attention networks, transformer, deep learning

## Abstract

Multivariate time series anomaly detection is a critical technique for industrial intelligent monitoring. However, existing methods often suffer from prohibitively high training costs and slow convergence, making them ill-suited for industrial scenarios that require frequent model retraining due to dynamic operating conditions. To this end, an efficient two-stage spatio-temporal attention detection framework, TSA-Net, is proposed. This framework adopts a two-branch architecture utilizing a structurally reparameterized temporal convolutional network (RepVGG-TCN) and a graph attention network (GAT). Crucially, the RepVGG design enhances feature extraction capability during training through a multi-branch structure while collapsing into a compact single-branch architecture for deployment, thereby optimizing structural complexity. At the core of TSA-Net is a cascading feedback mechanism, where preliminary predictions from the first stage serve as guidance signals to augment the input for the second stage, enabling coarse-to-fine iterative refinement. Furthermore, an adaptive gating mechanism dynamically fuses spatio-temporal features, improving the model’s adaptability. Extensive experiments with ten state-of-the-art algorithms on three benchmark datasets demonstrate that TSA-Net achieves significant optimization. Specifically, it improves the F1 score by approximately 7% while reducing the training time by up to 99% compared to complex Transformer-based models, offering a rapid-deployment solution for high-dimensional anomaly detection.

## 1. Introduction

With the rapid development of Industry 4.0 and Internet of Things (IoT) technologies, sensor data from industrial production lines, infrastructures, and complex devices is exploding. This data exists in the form of Multivariate Time Series (MTS), which records multi-dimensional state information of the system during operation. Real-time and accurate anomaly detection of these MTS data is of vital importance to ensure stable system operation, predict potential equipment failures, and avoid major economic losses and safety accidents. Consequently, there is an increased demand for industrial MTS anomaly detection methods. Early studies relied mainly on statistical or traditional machine learning models, but these approaches usually require extensive manual feature engineering and struggle to capture complex nonlinear relationships. In recent years, deep learning has shown great potential in this field due to its powerful automatic feature learning capability.

Models designed for sequence data were among the first to be applied to this task. Convolutional neural networks (CNNs) were used to extract localized patterns in time series. However, a significant weakness of standard CNN models is their inability to effectively capture long-range temporal dependencies [1]. Whereas Long Short-Term Memory Networks (LSTMs) and their variants (e.g., GRUs) excel in modeling global temporal dynamics through their recurrent structure, the Variable Segmentation LSTM (VLSTM) framework proposed in [2] is highly effective in dealing with industrial big data. However, recurrent networks inherently suffer from difficulties in parallelizing training and low computational efficiency. Temporal Convolutional Networks (TCNs) [3] combine causal and dilated convolution to retain the ability to capture long-range dependencies while enabling parallelizable computation. A notable trend is that TCNs are often combined with other architectures (e.g., Transformer) in MTS anomaly detection to capture multi-scale features [4]. Despite the progress made by these temporal-centric models, they share a common limitation: they focus primarily on the time dimension but largely ignore the spatially structured relationships among variables dictated by the physical topology of the system. Such approaches fail to adequately account for the contextual correlations between devices in industrial control systems (ICS).

To explicitly model the spatial dependencies between variables, Graph Neural Networks (GNNs) were introduced [5]. For instance, the GLIN framework [6] constructs a comprehensive representation of variable relationships by fusing local and global node information. To further optimize information aggregation, Graph Attention Networks (GATs) were introduced to assign different weights to neighbors [7]. However, modeling the spatial dimension alone is insufficient. An ideal anomaly detection model must understand both the spatial topology among variables and their dynamic evolution over time. To this end, recent research has focused on capturing dynamic spatio-temporal dependencies [8,9]. For example, MTAD [10] utilizes a shared graph attention module alongside prediction and reconstruction tasks. Similarly, GA-Tran [11] enhances the Transformer’s feature extraction by introducing multi-scale convolution. While these state-of-the-art methods achieve high detection accuracy, they often introduce significant computational complexity. Stacking multiple attention layers with complex graph structures dramatically increases the parameter count and computational overhead. Crucially, existing methods often suffer from prohibitive training costs and slow convergence, making them ill-suited for industrial scenarios that require frequent model retraining due to dynamic operating conditions. Although some researchers have turned to two-stage architectures to tackle these challenges, they often treat the stages as loosely coupled components, lacking a deep feedback mechanism to iteratively refine detection results.

To address the challenge of balancing high-dimensional modeling capability with training efficiency and model compactness, we propose an innovative two-stage spatio-temporal attention framework, TSA-Net. Unlike conventional paradigms that stack complex attention layers, TSA-Net employs a lightweight dual-branch architecture. We integrate a structurally reparameterized Temporal Convolutional Network (RepVGG-TCN) with a Graph Attention Network (GAT). The core motivation for introducing RepVGG is to decouple the training architecture from the inference architecture. This allows the model to benefit from a multi-branch structure’s rich feature extraction capability during training while collapsing into a compact single-branch structure during deployment, thereby optimizing structural complexity without sacrificing performance. Furthermore, we design a unique cascade feedback mechanism, where the initial prediction from the first stage serves as a prior knowledge signal to augment the input for the second stage, enabling iterative refinement of anomalies. An adaptive gating mechanism is also introduced to dynamically fuse spatio-temporal features based on their importance.

The main contributions of this work are summarized as follows:

- We propose a novel spatio-temporal feature extractor that integrates RepVGG-TCN and GAT. This design captures both intrinsic temporal dynamics and spatial correlations while ensuring model compactness and high training efficiency through structural reparameterization.
- We develop a unique cascade feedback mechanism that uses the initial prediction from the first stage to iteratively refine the input for the second stage, significantly improving the model’s sensitivity to subtle anomalies.
- We introduce an adaptive gated fusion strategy to dynamically weigh the importance of spatio-temporal features, enhancing the detection of composite anomalies.

The rest of the paper is organized as follows. Section 2 presents the related works. Section 3 details our proposed method. Section 4 demonstrates the efficacy of our method. Finally, conclusions are given in the last section.

## 2. Related Work

### 2.1. Temporal-Centric Modeling Approaches

A dominant technical line in time series anomaly detection focuses on modeling dependencies primarily in the time dimension, typically falling into reconstruction-based or prediction-based paradigms. Classical prediction-based methods using Recurrent Neural Networks (RNNs), such as LSTMs [2], effectively capture temporal dynamics but suffer from sequential computation bottlenecks. To improve training parallelism, architectures utilizing Temporal Convolutional Networks (TCNs) and Transformers [12] were introduced.

Within the reconstruction paradigm, notable two-stage frameworks have emerged. For example, TranAD [13] utilizes an adversarial training scheme with Transformers to amplify anomalies. However, its reliance on heavy self-attention mechanisms incurs high computational costs, making it less suitable for resource-constrained edge deployment compared to lightweight convolutional designs. Similarly, DTAAD [3] employs a TCN to capture multi-scale patterns in parallel. However, this parallel architecture processes features in isolated branches that are only fused at the final step. Unlike serial cascade frameworks that explicitly inject preliminary predictions back into the input to guide iterative refinement, DTAAD’s parallel independence prevents the model from correcting initial estimation errors effectively. Other hybrid models, such as the work by Cao et al. [14], combine Transformers and TCNs to leverage complementary features but often inherit the complexity of both. Consequently, these temporal-centric models typically treat sensors as independent channels, failing to explicitly model the complex spatial correlations dictated by industrial topologies.

### 2.2. Joint Spatio-Temporal Modeling Approaches

To capture inter-variable dependencies, joint spatio-temporal approaches have integrated Graph Neural Networks (GNNs) or attention mechanisms with sequence models. These methods can be broadly categorized based on their dependency modeling strategies.

**Static Graph and Attention-based Methods.** Mainstream strategies, such as MTAD-GAT [5] and ESTAD [15], combine graph attention or contrastive learning with GRUs to model correlations. To improve sequence modeling efficiency, works like MST-GAT [16] and STGaAN [17] integrate GAT with TCNs. However, these methods typically rely on static graph priors, which limit their adaptability to evolving operating conditions. Alternatively, attention-based methods like SiET [18] and the recent EST Transformer [19] utilize spatially enhanced Transformer architectures to capture global dependencies. While effective, standard multi-head attention often suffers from computational heaviness and potential subspace collapse, where diverse heads converge to redundant features.

**Dynamic Graph and Fine-grained Fusion.** To mitigate the limitations of predefined static topologies, recent research has gravitated towards dynamic dependency modeling. Approaches such as SP-GAT [9] and SSAD [20] employ sparse graph learning techniques to attenuate noise interference. Furthermore, D-GATAD [21] advances this paradigm by introducing a dynamic graph construction mechanism that updates the topological structure at each temporal step based on global information. Building on this, architectures like CST-GL [22] and ATCN [23] enhance detection precision through fine-grained graph fusion strategies, while ISOLATE [24] integrates these spatio-temporal frameworks into autoencoders to establish resilient representation learning capabilities.

**Advanced Adaptation and Interpretation Strategies.** Beyond standard architectural designs, researchers have explored auxiliary mechanisms to handle industrial complexity. For instance, Wu et al. [25] proposed a transfer learning system with cross-domain knowledge distillation to effectively address data distribution shifts, highlighting the critical role of adaptability in modern diagnostic systems. Simultaneously, emphasizing interpretability, Tang et al. [26] developed a gradient-based optimization method to explicate anomaly detection decisions. While these approaches significantly enhance system robustness and transparency, they typically necessitate additional computational modules or optimization steps. Consequently, integrating such sophisticated capabilities while maintaining the minimal inference latency required by resource-constrained edge devices remains an open challenge, motivating our focus on lightweight backbone design.

**Limitations.** Notwithstanding the superior detection performance achieved by the aforementioned methods, computational inefficiency persists as a significant constraint. Specifically, the continuous graph structure updates employed in D-GATAD [21] and the extensive self-attention mechanisms inherent to EST Transformer [19] impose substantial computational overheads. In industrial environments mandating rapid retraining and online adaptation, the elevated latency of such complex GNNs renders them suboptimal for real-time deployment. Consequently, there is a pressing need for architectures that effectively reconcile the trade-off between dynamic spatial modeling capabilities and inference efficiency.

### 2.3. Hierarchical Architectures and Computational Efficiency

Recent research has explored hierarchical frameworks to refine detection capabilities. Methods like MAD-STA [27] and GSTA-DeSVDD [28] utilize multi-stage designs with GATs or GANs. However, a key limitation of these approaches is loose coupling: they typically process intermediate features without explicitly leveraging the prior knowledge from the previous stage. Consequently, they lack a corrective mechanism to iteratively refine the hard-to-model patterns missed by the first stage.

Complementary to architectural complexity, computational efficiency constitutes a pivotal yet frequently overlooked objective. Although lightweight architectures have demonstrated efficacy in time series forecasting (e.g., LightTS [29]), contemporary anomaly detection paradigms predominantly depend on computationally intensive backbones to maintain detection precision. To bridge the gap between representational capacity and inference latency, structural reparameterization (e.g., RepVGG [30]), a technique characterizing the decoupling of multi-branch training topologies from streamlined single-branch inference architectures, has established its utility in computer vision. Nevertheless, the application of this methodology remains largely underexplored within the specific context of multivariate time series anomaly detection.

Summary: Existing methods struggle to balance three key aspects: spatial-temporal modeling depth, retraining efficiency, and deployment latency. TSA-Net addresses these gaps by: (1) Integrating a RepVGG-based architecture to achieve high training capacity and low inference latency; (2) Employing a serial cascade feedback mechanism with prior knowledge injection to enable deep iterative refinement, marking a departure from parallel paradigms such as DTAAD or loosely coupled frameworks; and (3) Utilizing an adaptive gated fusion to robustly handle noise in fully connected graphs.

## 3. Methodology

### 3.1. Problem Definition

This work addresses the problem of unsupervised anomaly detection for industrial Multivariate Time Series (MTS) data. Formally, a multivariate time series is represented as a finite-length sequence $X=\{x_{1},x_{2},\ldots ,x_{T}\}$, where *T* is the total length of the sequence. At any time step *t*, the observation $x_{t}\in R^{m}$ is an *m*-dimensional vector representing the readings of *m* different sensors or variables.

The objective is to compute a real-valued anomaly score $S_{t}$ for each time step *t* based on historical data. By comparing this score with a decision threshold τ, a binary anomaly label $y_{t}$ is determined:

(1) $$y_{t}=\left\{\begin{array}{cc} 1, & S_{t}>\tau \\ 0, & S_{t}\leq \tau \end{array}\right.$$

### 3.2. TSA-Net Framework

**Figure 1:**
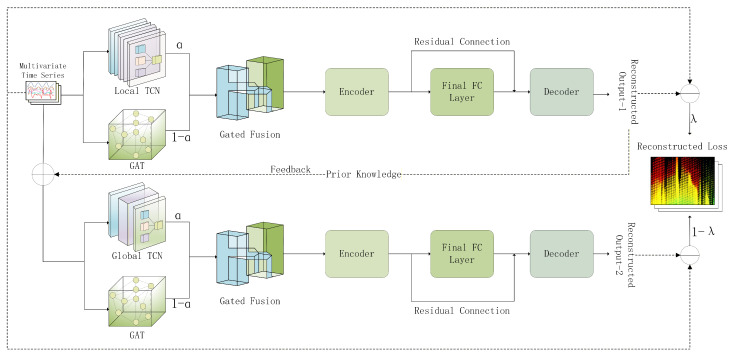
The overall architecture of the proposed TSA-Net framework. It adopts a two-stage design linked by a cascading feedback mechanism to enable coarse-to-fine iterative refinement.

The architecture of TSA-Net is illustrated in Figure 1. It adopts a two-stage design linked by a cascading feedback mechanism. In the first stage, parallel Local TCN and GAT branches capture temporal and spatial features, respectively, which are synthesized via Adaptive Gated Fusion and processed by an Encoder-Decoder to generate an initial reconstruction. Crucially, the preliminary predictions from this stage are fed back and added to the original input (⊕) to guide the second stage (utilizing a Global TCN and GAT) for iterative refinement. The entire network is optimized jointly via a weighted loss function balancing both stages.

#### 3.2.1. Spatio-Temporal Feature Extraction

To achieve a balance between high representational capacity and deployment efficiency, we propose RepVGG-TCN, an enhanced version of the standard Temporal Convolutional Network. The design incorporates structural reparameterization [31] to decouple the training architecture from the inference architecture.

**Figure 2:**
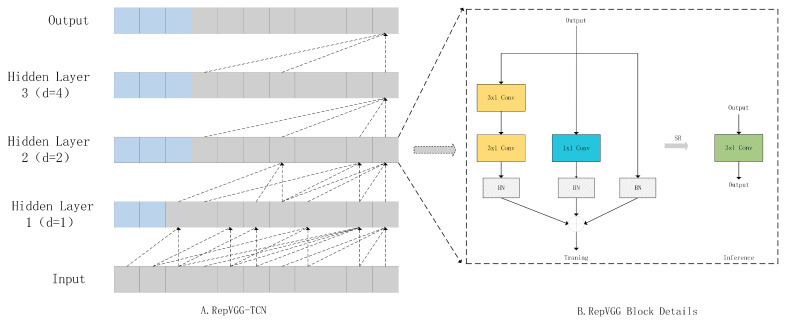
The architecture of the temporal feature extractor. (**A**) Structure of the Global TCN incorporating causal padding (blue blocks) and exponentially dilated convolutions. Note that the Local TCN adopts a congruent structure, with the specific distinction that the dilation factor is strictly fixed at d=1 across all layers. (**B**) Detailed mechanism of the RepVGG Block. During training, the module employs a multi-branch topology (incorporating 3×1 Conv, 1×1 Conv, and identity mapping) to enhance feature representation. During inference, these parallel branches are mathematically merged into a single 3×1 convolution via Structural Reparameterization (SR), thereby decoupling training complexity from inference latency.

As illustrated in Figure 2, during the training phase, a multi-branch architecture—incorporating a 3 × 1 convolution, a 1 × 1 convolution, and an identity mapping—is adopted. Theoretically, this topology facilitates efficient gradient propagation during backpropagation, effectively alleviating the vanishing-gradient phenomenon often inherent in deep temporal networks. This mechanism enhances the model’s capacity to capture long-range temporal dependencies. For the inference phase, these parallel branches are structurally reparameterized into a single 3 × 1 convolutional kernel. This transformation substantially minimizes memory-access costs and computational latency, thereby optimizing the model for deployment on resource-constrained industrial edge devices.

To ensure strict temporal causality, causal padding is applied:

(2) P=(K−1)×d

where *K* is the kernel size and *d* is the dilation factor. It is worth noting that the structural reparameterization is essentially a linear transformation of kernel weights, which does not alter the receptive field direction. By applying causal padding and Chomp1d operations strictly to all branches, we mathematically guarantee that the fused model maintains strict temporal causality, preventing any future information leakage. The total receptive field (TRF) satisfies:

(3) $$1+\left(K-1\right)\times \left(2^{L}-1\right)\geq R$$

where *L* is the number of layers, and *R* is the input sequence length. The minimum number of stacked layers required was determined to be 3 according to Equation (3). Therefore, all TCN modules in the proposed model consist of three convolutional layers with a convolutional kernel size of 3. The TCN operates in two modes: Local TCN (dilation d=1) for fine-grained patterns, and Global TCN (exponential dilation $d=2^{i}$) for long-range dependencies.

**Figure 3:**
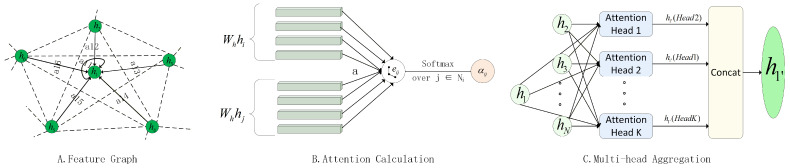
Schematic diagram of the spatial feature extractor (GAT). (**A**) Feature Graph Topology: Illustrates the spatial aggregation mechanism where a target node integrates information from its connected neighbors. (**B**) Attention Calculation: Details the computation of the pair-wise attention coefficient $e_{ij}$. This process utilizes the concatenation operation and a learnable weight vector a (Equation (4)) to dynamically quantify the importance of neighboring features. (**C**) Multi-head Aggregation: Depicts the fusion strategy where latent representations from *K* independent attention heads are concatenated to synthesize the final spatial embedding $h_{i}^{\prime }$.

Simultaneously, to model inter-variable dependencies, we construct a fully-connected attribute graph G=(V,E), where nodes represent sensors. A Graph Attention Network (GAT) captures dynamic spatial correlations, as shown in Figure 3. Given the input feature vectors $h=\{h_{1},h_{2},\ldots ,h_{N}\}$, the attention coefficient $e_{ij}$ between node *i* and its neighbor *j* is computed as:

(4) $$e_{ij}=\mathrm{LeakyReLU}\left(a^{T}\left[Wh_{i}∥Wh_{j}\right]\right)$$

where W is a learnable weight matrix for linear transformation, a is a learnable weight vector for the attention mechanism, and ‖ denotes the concatenation operation. Normalized attention weights $\alpha_{ij}$ are obtained via the softmax function:

(5) $$\alpha_{ij}=\frac{\exp(e_{ij})}{\sum_{k\in N_{i}}\exp\left(e_{ik}\right)}$$

where $N_{i}$ represents the set of neighbor nodes for node *i*.

To stabilize the learning process and capture the dependencies of different aspects, a multi-head attention mechanism is employed. The core strategy involves executing *K* independent attention computations in parallel, where the updated feature vector $h_{i}^{\prime }$ for node *i* within each head is obtained by a weighted aggregation of its neighboring features, as calculated below:

(6) $$h_{i}^{\prime }=\mathrm{ReLU}\left(\sum_{j\in N_{i}}\alpha_{ij}Wh_{j}\right)$$

Subsequently, the resulting *K* feature vectors, denoted from ${h^{\prime }}_{i}^{\left(1\right)}$ to ${h^{\prime }}_{i}^{\left(K\right)}$, are concatenated to form a richer and more powerful final representation $H_{i}$ [16]:

(7) $$H_{i}=\mathrm{Concat}\left({h^{\prime }}_{i}^{\left(1\right)},{h^{\prime }}_{i}^{\left(2\right)},\ldots ,{h^{\prime }}_{i}^{\left(K\right)}\right)$$

Despite the fully connected initialization, the attention mechanism functions as an adaptive soft-sparsification filter. By driving the attention coefficients of irrelevant sensor pairs toward zero ($\alpha_{ij}\approx 0$), the model effectively suppresses noise propagation from uncorrelated sensors, thereby approximating the underlying sparse topology.

#### 3.2.2. Adaptive Gated Fusion

To dynamically integrate temporal features T and spatial features G, we introduce an adaptive gating mechanism. First, features are concatenated along the channel dimension:

(8) $$C=\mathrm{Concat}([T,G],\dim=1)\in R^{B\times 2F\times W}$$

where dim=1 is the feature splicing dimension, *B* is the batch size, *F* is the number of features, and *W* is the sequence length. Next, a one-dimensional convolutional network (Conv1d) and a Sigmoid activation function are utilized to process the combined features C so as to dynamically generate a fusion gating weight for each time step and feature dimension A. Setting the convolutional kernel size to 1 ensures that the weights for each time step are computed independently, which enables efficient computation while also utilizing the parameter-sharing advantage of convolution. The definition is as follows:

(9) $$A=\mathrm{Sigmoid}(\mathrm{Conv}1d(C))\in R^{B\times F\times W}$$

Finally, the learned gating weights A are used to weight and sum the TCN feature T and GAT feature G to obtain the final fusion feature $F^{\prime }$:

(10) $$F^{\prime }=A⊙T+\left(1-A\right)⊙G$$

where ⊙ denotes element-wise multiplication.This mechanism allows the model to adaptively emphasize either temporal or spatial information at each time step.

#### 3.2.3. Encoder-Decoder

**Figure 4:**
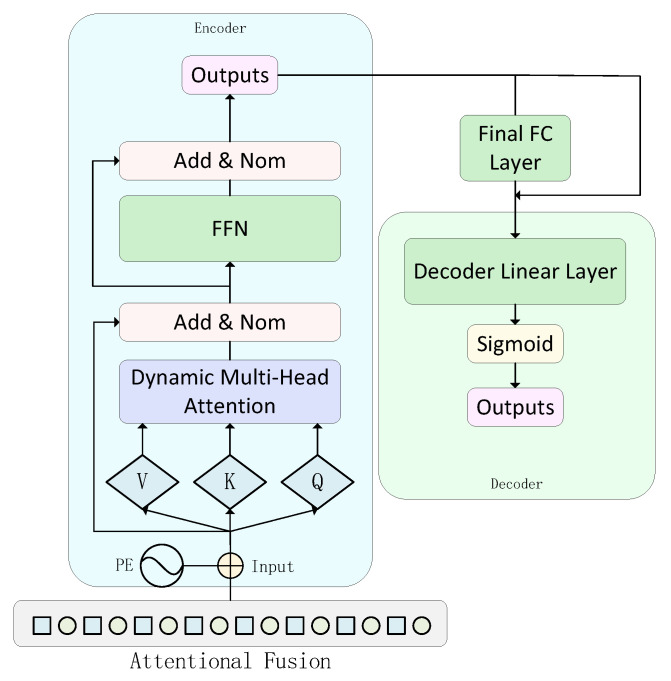
The Encoder-Decoder architecture.

To further capture the long-range temporal dependencies in the fused features, the architecture of TSA-Net integrates a lightweight Transformer-based encoder-decoder module, as shown in Figure 4, with its encoder core components including:

Adaptive Multi-Head Self-Attention: The standard Transformer uses a fixed number of heads *h*. To improve the adaptivity and efficiency of the model, a dynamic head-counting strategy is proposed. Specifically, the number of heads *h* is no longer a fixed hyperparameter but is set dynamically according to the dimension *D* of the input features: *h* is set as the largest divisor of *D* other than 1. If *D* is prime, *h* is set to *D*. This strategy aims to automatically match computational resources for features of different complexity, which significantly reduces redundant computations while ensuring the expressive power of the model. In the proposed adaptive multi-head attention mechanism, the underlying computation of each attention head follows the standard scaled dot product attention model [8]:

(11) $$\mathrm{Attention}\left(Q,K,V\right)=\mathrm{softmax}\left(\frac{QK^{T}}{\sqrt{d_{k}}}\right)V$$

where *Q*, *K*, and *V* denote the Query, Key, and Value matrices respectively, and $d_{k}$ is the dimension of *Q* and *K*.

Modified Feedforward Network: The standard position-independent feedforward network (FFN) is fine-tuned after the output *X* of the attention sublayer. LeakyReLU is adopted as the activation function, aiming to enhance the model’s responsiveness to weak nonlinear anomalous signals commonly found in industrial scenarios, as defined below:

(12) $$\mathrm{FFN}\left(x\right)=\mathrm{LeakyReLU}\left(xW_{1}+b_{1}\right)W_{2}+b_{2}$$

where $\left(W_{1},b_{1}\right)$, $\left(W_{2},b_{2}\right)$ are the weights and bias terms of the first and second fully connected layers, respectively.

After obtaining the preliminary coded representation, an additional Fully Connected (FC) layer with residual connectivity is introduced in order to enhance the sensitivity to weak anomalies, augmenting the preliminary representation to obtain the final coded representation. Finally, a lightweight decoder consisting of a single linear layer and a Sigmoid activation function is used to map the final encoded representation back to the input dimension to generate the prediction ${\hat{X}}^{\left(i\right)}$.

#### 3.2.4. Cascade Feedback

To improve the model’s detection accuracy for complex and weak anomalies, a two-stage cascaded prediction feedback framework is designed. Instead of feeding the prediction error (residual) which might introduce noise, we adopt a Prior Knowledge Injection. The core idea of the framework is to model multi-stage prediction as an iterative refinement [32] and coarse-to-fine [33] process. The first-stage network generates a preliminary prediction that serves as a coarse-grained trend estimator. This estimator serves as explicit prior knowledge and is injected back into the input space to guide the second stage. By explicitly superimposing the preliminary prediction onto the original input, the second-stage network is provided with prior knowledge of the temporal trends, allowing it to focus on finer judgment and correction of the preliminary prediction signals rather than learning the global pattern from scratch.

Specifically, instead of directly conveying the first-stage prediction result ${\hat{X}}^{\left(1\right)}$ as an isolated message, it is regarded as a guidance signal to the original input X. To this end, ${\hat{X}}^{\left(1\right)}$ is first reshaped to match the dimensions of the input and then added element-by-element with the original input *X* to form the augmented input $X^{\prime }$ in the second stage, which is defined as follows:

(13) $$X^{\prime }=X+{\hat{X}}_{\mathrm{reshaped}}^{\left(1\right)}=X+\mathrm{permute}({\hat{X}}^{\left(1\right)},\left(0,2,1\right))$$

where $\mathrm{permute}({\hat{X}}^{\left(1\right)},\left(0,2,1\right))$ denotes a dimension permutation that exchanges the time and feature dimensions.

The explicit superimposition of the preliminary prediction onto the input provides the second-stage network with a direct reference to the underlying temporal trend. Mathematically, this operation constitutes a Prior Knowledge Injection. Consequently, the second stage is relieved from the burden of learning feature representations ab initio; instead, it focuses on refining the fused representation comprising both the raw data and the estimated trend. Furthermore, this mechanism establishes an efficient gradient flow from the second stage back to the input, facilitating cooperative learning and significantly accelerating convergence compared to decoupled training paradigms.

Upon receiving the augmented input *X*′, it is fed into a processing flow with the same structure as the first stage to output the final, more accurate prediction ${\hat{X}}^{\left(2\right)}$. This end-to-end cascade design enables the model to construct an efficient self-correction loop, which improves the accuracy and robustness of the overall detection task.

### 3.3. Training Process

#### 3.3.1. Objective Function

To achieve end-to-end optimization of the two-stage cascaded feedback framework, a jointly supervised objective function is used. This function co-optimizes the two decoders by combining their respective losses, assigning a higher priority to the first stage’s prediction task via a fixed hyperparameter λ.

Specifically, the initial prediction loss $L_{1}$ in the first stage and the prediction loss $L_{2}$ in the second stage, which incorporates the injected prior knowledge, are calculated separately. These two mean square error (MSE)-based losses are weighted and combined to form the final optimization objective [34]:

(14) $$L\left(\Theta_{1},\Theta_{2}\right)=\lambda L_{1}+\left(1-\lambda \right)L_{2}$$

where $\Theta_{1}$ and $\Theta_{2}$ denote the full learnable parameters of the two-stage encoder-decoder, respectively. In this work, we set λ=0.8. This assigns a higher weight to the first stage to ensure the model learns a robust coarse-grained foundation, which stabilizes the subsequent iterative refinement.

#### 3.3.2. Anomaly Scoring and Thresholding

During inference, the anomaly score for each feature is computed as the weighted sum of squared prediction errors from both stages, ensuring sensitivity to both the global trend deviations captured in the first stage and the subtle anomalies refined in the second stage. To obtain a system-level anomaly judgment, we adopt a mean-aggregation logic. Specifically, the global anomaly score $S_{t}$ at time *t* is the average of scores across all *m* dimensions:

(15) $$S_{t}=\frac{1}{m}\sum_{j=1}^{m}s_{t,j}$$

where $s_{t,j}$ is the anomaly score of the *j*-th feature. We adopt this mean-aggregation strategy to ensure robustness against single-sensor noise, which is prevalent in high-dimensional industrial data. Unlike max-aggregation, which is hypersensitive to transient spikes in unrelated sensors (leading to false positives), averaging effectively suppresses independent noise while highlighting synergistic deviations that propagate across multiple correlated variables. This aligns with the physical nature of system-level faults, where anomalies typically manifest as collective disturbances.

The decision threshold is determined adaptively using the Peaks-Over-Threshold (POT) method based on Extreme Value Theory (EVT) [35]. This statistical approach fits a Generalized Pareto Distribution (GPD) to the tail of the score distribution to find a robust threshold ${\mathrm{thr}}_{\mathrm{POT}}$. The final anomaly label $y_{t}$ is:

(16) $$y_{t}=\left\{\begin{array}{cc} 1 & \mathrm{if}\;S_{t}\geq {\mathrm{thr}}_{\mathrm{POT}}\\ 0 & \mathrm{otherwise} \end{array}\right.$$

This aggregation strategy effectively reduces false alarms compared to strict intersection strategies while maintaining high sensitivity to system-wide anomalies.

## 4. Experiments

### 4.1. Dataset Description

The statistical details of these datasets are summarized in Table 1. The table provides an overview of the number of dimensions, entities, the size of the training and testing sets, and the percentage of anomalies. Specifically, the ‘Dimensions’ column follows the format of Entities (Features). For instance, the SMD dataset contains data from four distinct server machines (entities), each monitored across 38 features.

- Soil Moisture Active Passive (SMAP): This dataset is a 25-dimensional publicly available dataset collected by NASA [9] and contains telemetry information and anomaly data extracted from Anomalous Event Anomaly (ISA) reports from spacecraft monitoring systems.
- Mars Science Laboratory (MSL): This dataset is similar to SMAP and contains actuator and sensor data from the Mars Rover itself [5].
- Server Machine Dataset (SMD): This dataset is a newly collected 5-week-long dataset from a large internet company and is divided into two equally sized subsets of training and testing sets [13].

### 4.2. Data Preprocessing

The following preprocessing steps were applied to all datasets:

- Normalization: To address varying scales across features, Min-Max normalization is applied to all datasets, mapping the values to the interval [0, 1].
- Noise Enhancement: Gaussian white noise is added to the datasets to enhance model robustness.
- Sliding Window Processing: Slicing the original time series with a sliding window of size *w* converts it into a dataset of overlapping, fixed-length subsequences, which enables the model to process the data efficiently.

### 4.3. Evaluation Metrics

Considering the prevalence of data imbalance in anomaly detection tasks, model performance is evaluated using multiple metrics. Precision, Recall, and their harmonic mean, the F1-score, are used to measure the accuracy and coverage of the model’s predictions. Additionally, the Area Under the Curve (AUC) is used to assess the overall classification performance and robustness under various decision thresholds.

### 4.4. Baseline Methods

The performance of TSA-Net is benchmarked against ten state-of-the-art anomaly detection methods:

- LSTM-NDT [36]: A model employing a unique dual LSTM parallel structure. Aims to capture different levels of time series dependence through two parallel recurrent neural networks.
- DAGMM [37]: A classical approach combining deep autoencoders and Gaussian mixture models.
- OmniAnomal [38]: By sampling from a probability distribution and decoding the reconstruction, the model better captures the randomness and dynamics of normal data and detects anomalies by reconstructing the probabilities.
- MAD-GAN [39]: A reconstruction method based on GAN networks to compute anomaly scores and capture temporal correlation of time series distributions using LSTM-RNN.
- MTAD-GAT [5]: A model that combines graph attention networks and recurrent neural networks. After capturing these complex dependencies via GAT, the enhanced feature representation is fed into a GRU network to model temporal dynamics.
- USAD [40]: Designed an encoder and two independent decoders to form two competing self-encoders. During training, one self-encoder tries to minimize the reconstruction error, while the other tries to distinguish between the real data and the other’s reconstructed data.
- GDN [41]: It is proposed to learn relationships between sensors in the form of graphs and then recognize and interpret deviations from the learned patterns.
- CAE-M [42]: A convolutional autoencoder that treats the time series window as a 2D matrix and uses standard convolutional and transpositional convolutional layers for encoding and decoding, focusing on its local feature extraction capabilities of convolutional operations to learn patterns in normal data and detecting anomalies through reconstruction errors.
- TranAD [13]: Advanced Transformer-based models utilize the idea of enlarging the error to effectively amplify anomalous signals, and direct their internal attention mechanisms by reconstructing the error-derived focus scores to make the model more sensitive to deviations.
- DTAAD [3]: Proposes an autoregressive-autoencoder (AR-AE) framework that extracts long and short-term dependencies separately via a parallel two-branch TCN combined with a Transformer and utilizes a callback mechanism to amplify the reconstruction error to enhance the detection of weak anomalies.

### 4.5. Hyperparameters

**Figure 5:**
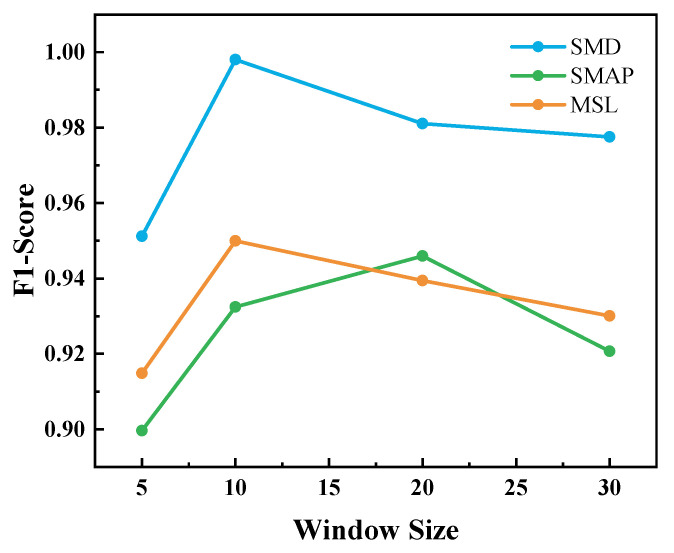
F1 score with different window size.

Our model was implemented using the PyTorch framework (version 2.0.0, Linux Foundation, San Francisco, CA, USA) with CUDA (version 11.7, NVIDIA Corporation, Santa Clara, CA, USA). The programming language used was Python (version 3.8, Python Software Foundation, Wilmington, DE, USA). The model was trained on a server equipped with an Intel Core i5-9400F CPU (Intel Corporation, Santa Clara, CA, USA), 16 GB of RAM, and a single NVIDIA A40 GPU (NVIDIA Corporation, Santa Clara, CA, USA). The Adam optimizer was used for model optimization. The initial learning rate was set to 0.001 for the SMD and SMAP datasets, and 0.002 for the MSL dataset, with a weight decay of $10^{-5}$ applied across all datasets. A StepLR learning rate scheduler was used with a step size of 5 and a decay rate of 0.9. A batch size of 128 was used for all training procedures. As illustrated in Figure 5, a window size of 10 was selected, as it yielded optimal F1 scores.

In terms of model architecture, the TCN module has a convolutional kernel size set to 3 and a Dropout rate of 0.2. To ensure the training stability of the multi-branch RepVGG structure on noisy industrial data, we adopted Kaiming Initialization (He Normal) for all convolutional weights. This strategy aligns with the LeakyReLU activation function to maintain consistent gradient variance, effectively preventing gradient explosion or vanishing during the early training phase. The GAT module uses 2 attention heads with a Dropout rate of 0.2 and constructs an adaptive complete graph. The encoder consists of a single layer. The number of attention heads is dynamically determined as described in Section 3.2.3. We set the number of hidden units to 16, the number of feedforward layers to 2, and the Dropout rate to 0.1. The gated fusion mechanism uses a convolutional kernel of size 1. For the threshold determination of anomaly scores, we employ the Peak-Over-Threshold (POT) method across all comparative models to ensure a fair evaluation. Specifically, the Generalized Pareto Distribution (GPD) parameters are estimated using the peaks (excesses) above an initial threshold. This threshold is calibrated using the empirical quantile of the training anomaly scores (e.g., the top 2% quantile for the SMAP dataset). Subsequently, the final detection level *q* is set to $1\times 10^{-5}$ to derive the dynamic threshold ${\mathrm{thr}}_{\mathrm{POT}}$.

### 4.6. Performance Comparison

The performance of TSA-Net is comprehensively compared with ten state-of-the-art methods on three publicly available benchmark datasets, and the detailed results are presented in Table 2. To ensure experimental reliability, the results for TSA-Net are reported as the average over five independent runs. For baseline methods, our reproduced results are consistent with the performance reported in the original literature.

The quantitative analysis demonstrates that TSA-Net exhibits superior performance on key metrics. Specifically, in terms of the F1-score, TSA-Net achieves the state-of-the-art performance on both the SMD and SMAP datasets, recording scores of 0.9782 and 0.9297, respectively. Notably, on the SMD dataset, TSA-Net significantly outperforms the second-best method (USAD, 0.9493), highlighting its capability to handle complex anomalies. On the MSL dataset, TSA-Net achieves a competitive F1-score of 0.9389. Although this is slightly lower than the best baseline model, GDN (0.9588), TSA-Net remains comparable to other top-tier methods such as TranAD and DTAAD.

Furthermore, TSA-Net maintains high AUC values across all three datasets (0.9865 on SMD, 0.9945 on SMAP, and 0.9892 on MSL). These consistent results indicate that the proposed model achieves a strong balance between precision and recall, effectively minimizing false alarms while detecting anomalies. Collectively, the high mean performance with low variance (as detailed in the table footer) underscores the robustness and effectiveness of TSA-Net in diverse multivariate time-series anomaly detection scenarios.

### 4.7. Computational Efficiency Analysis

To comprehensively validate the Rapid Deployment capability of TSA-Net in industrial scenarios, we conducted a rigorous evaluation covering both the training phase (convergence speed) and the inference phase (deployment latency).

#### 4.7.1. Training Efficiency and Convergence

We evaluated the computational efficiency based on training time per epoch and Total Convergence Time across all methods. The Total Convergence Time is strictly defined as the accumulated wall-clock training time required to reach the epoch where the model achieves its peak F1-score on the test dataset. As detailed in Table 3, TSA-Net exhibits significant advantages. Not only does it reduce the per-epoch training time by up to 99% compared to complex GNN-based models (e.g., MTAD-GAT), but it also demonstrates superior convergence speed. Specifically, on the large-scale SMD dataset, TSA-Net achieves convergence in just 545 s, which is approximately 80× faster than MTAD-GAT (45,600 s) and 3× faster than the efficient Transformer-based TranAD (905 s). This exceptional efficiency enables rapid model retraining and adaptation to dynamic industrial environments, minimizing downtime during system updates.

#### 4.7.2. Inference Latency and Deployment

To further validate the feasibility of edge deployment, we evaluated the inference speed on a CPU environment (Intel Core i5) with a batch size of 1, simulating real-time data streaming. The quantitative results are presented in Table 4.

At the module level, the structural reparameterization demonstrates its core value: the fused TCN module reduces latency from 0.79 ms to 0.34 ms, achieving a remarkable 2.31× speedup. This confirms that the decoupling strategy effectively eliminates redundant memory access costs during inference. At the system level, while inference latency is partially constrained by the graph computation, the optimization of the temporal branch ensures that the fully fused TSA-Net achieves an end-to-end latency of 4.35 ms per sample. This supports a throughput of ∼230 samples/s, which comfortably exceeds the data sampling rates of most industrial SCADA systems (typically 1–100 Hz), verifying the model’s capability for real-time anomaly monitoring.

#### 4.7.3. Training Dynamics and Convergence

**Figure 6:**
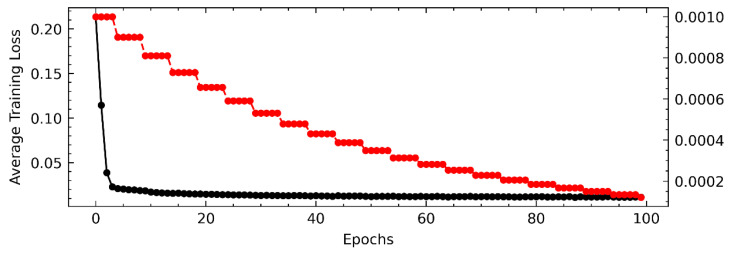
Training process analysis on the SMAP dataset. The black curve represents the Average Training Loss (refer to the left y-axis), while the red curve indicates the Learning Rate schedule (refer to the right y-axis). The regular stepwise descent of the red curve reflects the pre-defined StepLR strategy, which helps the model effectively escape local minima and stabilize convergence.

Figure 6 illustrates the training dynamics using a dual-axis plot. The average training loss (black curve, left axis) decreases rapidly within the first 60 epochs, demonstrating fast convergence. The observed stepwise descent pattern (red curve, right axis) corresponds to the pre-defined learning rate decay schedule (StepLR), confirming that the optimizer effectively escapes local minima at each stage.

### 4.8. Visualization Analysis

#### 4.8.1. Spatio-Temporal Correlation Analysis

**Figure 7:**
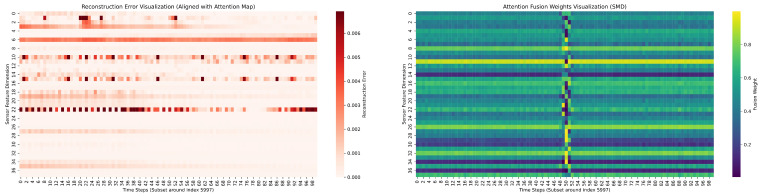
Visualization of Reconstruction Error and Attention Fusion Weights around an anomaly event (index 5997). The dark regions in the attention map demonstrate the model’s soft-sparsification capability during normal operation, while the bright band indicates the macro-level isolation mechanism engaging to prevent noise propagation during anomalies. The x-axis represents time steps, and the y-axis represents sensor features.

To demonstrate how TSA-Net captures spatial correlations and adapts to anomalies, we visualize the Attention Fusion Weights and the corresponding Reconstruction Errors in Figure 7. As shown in the reconstruction error map, a system-wide anomaly emerges around time steps 49–52. Simultaneously, the attention fusion weight map reveals a distinct regime shift. These visual patterns elucidate the critical contribution of the spatial (GAT) module and provide empirical validation for the proposed strategy to mitigate noise propagation inherent in fully connected graph initializations:

**Soft-Sparsification and the Cornerstone of Normalcy (Dark regions):** For the vast majority of the timeline (normal periods), the fusion weights remain low (blue/green), indicating that the model actively leverages the GAT module. This refutes the assumption that a fully connected initialization implies uniform dependency. Instead, the GAT’s attention mechanism performs soft-sparsification Equation (5) by learning to assign negligible weights to irrelevant sensor pairs. This allows the model to extract a sparse, structured latent topology from the fully connected graph, effectively filtering out noise while capturing precise physical dependencies (e.g., cause-and-effect) that are crucial for defining normal system behavior.

**Macro-level Isolation against Noise Propagation (Bright band):** When the anomaly strikes, the physical consistency between sensors is often disrupted, turning the graph structure into a source of noise. The sudden shift to high fusion weights (≈1, bright yellow) indicates that the Adaptive Gating mechanism acts as a macroscopic safety valve. It detects this context violation and temporarily isolates the spatial branch. This mechanism prevents the error propagation caused by the breakdown of spatial correlations, forcing the model to rely on intrinsic temporal trends (TCN) for robust reconstruction during the crisis.

In summary, TSA-Net solves the fully connected noise problem hierarchically: it uses attention to prune edges at the micro-level during stable operation, and uses gating to prune the entire branch at the macro-level during systemic failures. This synergy ensures that GAT serves as a precise baseline for normalcy without compromising robustness.

#### 4.8.2. Case Study

**Figure 8:**
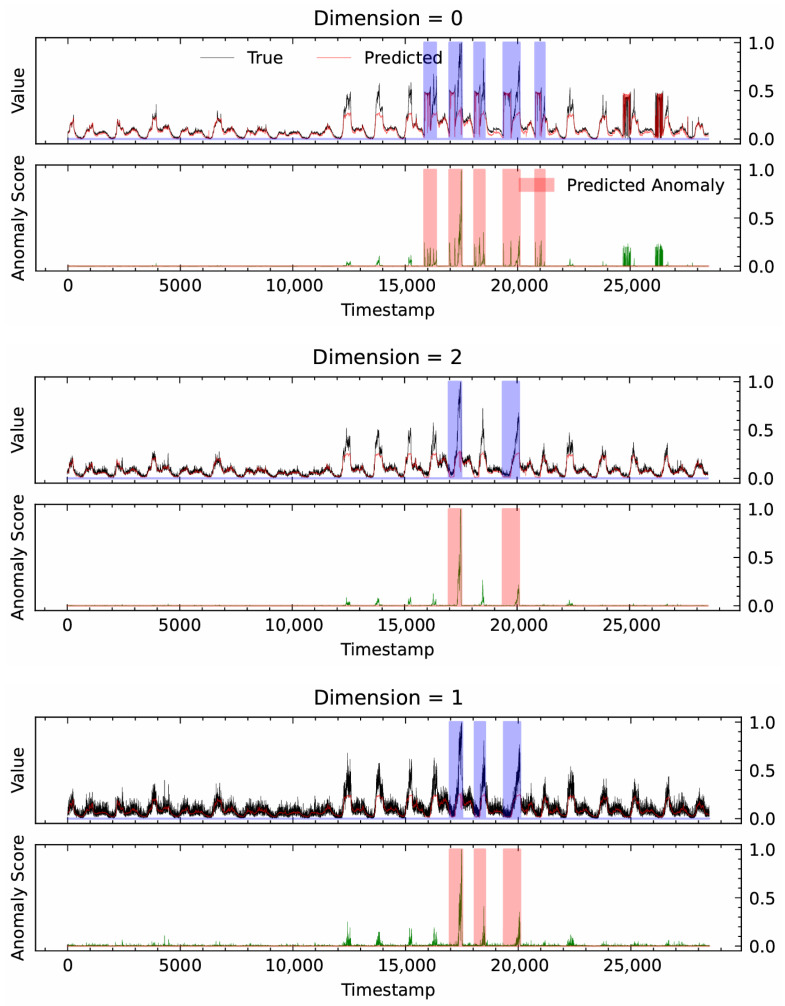
Qualitative evaluation of the model’s performance on the first three dimensions of the SMD dataset. The figure visualizes the relationship between the ground truth data (black line), model predictions (red line), and anomaly scores (green line). The **purple-shaded regions** represent the ground truth anomaly intervals, while the **red-shaded regions** indicate the anomalies detected by the model.

Figure 8 provides a qualitative evaluation of the model’s performance on the first three dimensions of the SMD dataset, visualizing the relationship between ground truth data, model predictions, and anomaly scores. During normal operational periods (non-shaded regions), a model’s predictions (red line) closely track the ground truth (black line). This indicates that the model has effectively learned the normal temporal patterns, as evidenced by the correspondingly low and stable anomaly scores (green line).

Conversely, within the ground truth anomaly intervals, such as the purple-shaded region from timestamp 17,000 to 21,000, the data exhibits significant deviations from the established pattern. The model’s predictions, however, maintain the learned normal trend, creating a substantial discrepancy that leads to a sharp increase in the anomaly score. Once this score surpasses a predefined threshold, the segment is correctly identified as an anomaly (red-shaded region). A high degree of overlap is observed between the detected and ground truth anomalies, a finding that is consistent with the 0.9782 F1-score reported in Table 2 and thus corroborates the model’s effectiveness.

A minor deviation is noted in dimension 0 (post-timestamp 25,000), where the model flags a potential anomaly not present in the ground truth labels. This event could be interpreted as a false positive, reflecting the model’s high sensitivity to subtle fluctuations, or it may indicate an unlabeled micro-anomaly within the dataset. Overall, the multi-dimensional analysis demonstrates the model’s robustness and its capability to reliably capture anomalous events, thereby enhancing the credibility of the detection results.

### 4.9. Ablation Studies

To further validate the effectiveness of each component in the TSA-Net architecture, systematic ablation experiments were conducted on three benchmark datasets. The results are presented in Table 5. By gradually removing key components, the specific contribution of each module to the overall performance is quantitatively analyzed. When the Local-STF module was removed (i.e., relying only on the global processing stage), the F1 scores on SMD, SMAP, and MSL dropped to 0.9660, 0.8714, and 0.8896, respectively. This highlights the importance of local feature extraction for capturing fine-grained patterns. Conversely, removing the Global-STF module (composed of global TCN and GAT) caused the performance to drop to 0.9679, 0.8735, and 0.8957, demonstrating that global information integration is crucial for performance improvement. After removing the feedback connection, the F1 scores became 0.9731, 0.9072, and 0.8458, with a significant decrease on the MSL dataset in particular, demonstrating the key role of the feedback mechanism in information transfer and feature enhancement. Removing the Transformer component led to a substantial performance drop to 0.9582, 0.8869, and 0.7806, highlighting its important role in capturing long-range sequence dependencies. Finally, using simple summation fusion instead of the gated fusion mechanism resulted in F1 scores of 0.9679, 0.8676, and 0.8416, which demonstrates the significant advantages of the proposed gated fusion mechanism in effectively integrating different features. Furthermore, to verify the necessity of the structural reparameterization strategy, we replaced the RepVGG-TCN block with a standard single-branch TCN; the performance degraded consistently across all datasets, with F1-scores dropping to 0.9655 on SMD, 0.8812 on SMAP, and 0.8530 on MSL. This decline indicates that the multi-branch topology prevents gradient degradation and enables the model to capture finer temporal patterns than a standard TCN.

The ablation study validates the effectiveness of each component in TSA-Net. The synergy between these modules contributes to the model’s superior performance, confirming the rationality of the proposed architectural design.

To validate the effectiveness of the dynamic head-counting rule, we compared the proposed setting (h=19) with other configurations on the SMD dataset (D=38). As shown in Table 6, the proposed strategy yielded the lowest Test MSE of 0.0025, outperforming both the single-head baseline (h=1, MSE 0.0030) and the fully-split extreme case (h=38, MSE 0.0031). This confirms that the strategy effectively maximizes feature diversity without inducing subspace collapse.

## 5. Conclusions

In this paper, we propose TSA-Net, a lightweight two-stage spatio-temporal attention network for multivariate time series anomaly detection. To address the conflict between complex modeling and deployment efficiency, TSA-Net integrates a structurally reparameterized Temporal Convolutional Network (RepVGG-TCN) with a Graph Attention Network (GAT). This design successfully decouples the training architecture from the inference architecture, ensuring rich feature extraction capability during training while maintaining a compact model structure for deployment. Furthermore, a unique cascading feedback mechanism is introduced to utilize preliminary prediction signals for guided iterative refinement, alongside an adaptive gated fusion strategy that dynamically balances spatio-temporal dependencies.

Experimental results on three public benchmark datasets (SMD, SMAP, and MSL) demonstrate that TSA-Net outperforms state-of-the-art methods, improving the F1 score by approximately 7%. Crucially, the model reduces per-epoch training time by up to 99% compared to complex baselines. This exceptional training efficiency makes TSA-Net a practical solution for industrial scenarios requiring frequent model retraining and rapid adaptation to dynamic operating conditions.

In future work, we plan to address the computational bottleneck introduced by the full-graph construction in the spatial module. We aim to explore sparse graph learning or graph sampling techniques to further improve inference throughput in ultra-high-dimensional environments, extending the applicability of TSA-Net to larger-scale real-world systems.

## Figures and Tables

**Table 1:** Dataset characteristics and anomaly ratios.

Dataset	Dimensions	Train	Test	Anomalies (%)
SMD	38 (4)	708,420	708,420	4.16
SMAP	25 (55)	135,183	427,617	13.13
MSL	55 (3)	58,317	73,729	10.72

**Table 2:** Performance comparison with state-of-the-art methods. TSA-Net results are reported as the average over 5 independent runs to ensure stability. Best results are in bold.

Method	SMD					SMAP					MSL			
	P	R	AUC	F1		P	R	AUC	F1		P	R	AUC	F1
LSTM-NDT	0.9734	0.8430	0.9669	0.9034		0.8523	0.7317	0.8600	0.7874		0.6288	0.9991	0.9530	0.7721
DAGMM	0.9103	0.9905	0.9952	0.9488		0.8067	0.9882	0.9883	0.8874		0.7363	0.9992	0.9714	0.8482
OmniAnomaly	0.8879	0.9975	0.9944	0.9395		0.8128	0.9409	0.9887	0.8723		0.7846	0.9915	0.9780	0.8762
MAD-GAN	0.9981	0.8431	0.9931	0.9142		0.8157	0.9207	0.9889	0.8655		0.8516	0.9921	0.9860	0.9166
MTAD-GAT	0.8210	0.9208	0.9921	0.8681		0.7993	0.9984	0.9845	0.8880		0.7917	0.9817	0.9889	0.8762
USAD	0.9062	0.9967	0.9933	0.9493		0.7480	0.9619	0.9889	0.8412		0.7948	0.9904	0.9794	0.8817
GDN	0.7170	0.9965	0.9924	0.8340		0.7482	0.9884	0.9864	0.8521		0.9308	0.9885	0.9814	**0.9588**
CAE-M	0.9080	0.9662	0.9781	0.9360		0.8193	0.9559	0.9901	0.8824		0.7753	0.9991	0.9903	0.8735
TranAD	0.9052	0.9964	0.9932	0.9487		0.8103	0.9989	0.9886	0.8950		0.9036	0.9990	0.9914	0.9492
DTAAD	0.8464	0.9966	0.9891	0.9152		0.8221	0.9991	0.9910	0.9026		0.9037	0.9991	0.9917	0.9494
**TSA-Net**	0.9795	0.9769	0.9865	**0.9782**		0.8724	0.9952	0.9945	**0.9297**		0.8950	0.9873	0.9892	0.9389

Note: To verify the robustness of the proposed method, we conducted experiments with 5 random seeds. The values in the table represent the Mean performance. The corresponding standard deviations (Mean ± Std) for TSA-Net are as follows: SMD: P (0.9795 ± 0.0185), R (0.9769 ± 0.0210), AUC (0.9865 ± 0.0150), F1 (0.9782 ± 0.0201). SMAP: P (0.8724 ± 0.0192), R (0.9952 ± 0.0035), AUC (0.9945 ± 0.0042), F1 (0.9297 ± 0.0146). MSL: P (0.8950 ± 0.0145), R (0.9873 ± 0.0085), AUC (0.9892 ± 0.0075), F1 (0.9389 ± 0.0124).

**Table 3:** Comparison of computational efficiency. The table reports the training time per epoch and the total time to convergence on three datasets. TSA-Net achieves the fastest training speed.

	Time per Epoch (s)				Total Convergence Time (s)		
Method	SMD	SMAP	MSL		SMD	SMAP	MSL
LSTM-NDT	57.63	25.52	24.25		10,373	4441	4074
DAGMM	31.59	8.68	8.28		1011	278	314
OmniAnomaly	35.39	17.31	14.26		2760	1264	984
MAD-GAN	30.14	15.71	15.63		1356	597	594
MTAD-GAT	1128.82	157.11	198.73		45,600	4713	5563
USAD	58.19	5.71	5.14		2036	348	288
GDN	144.92	10.51	17.43		4203	736	1134
CAE-M	517.13	41.34	120.21		10,922	1984	3607
TranAD	22.59	15.71	16.27		905	628	520
DTAAD	21.27	1.51	2.81		894	105	78
TSA-Net	**13.63**	**1.48**	**1.19**		**545**	**59**	**38**

**Table 4:** Inference efficiency analysis on an edge-simulated CPU environment (Intel Core i5, Batch Size = 1). The structural reparameterization (Fusion) significantly reduces latency.

Scope	Structural Topology	Latency (ms)	Speed-Up	Throughput (Samples/s)
TCN Module	Multi-branch (Unfused)	0.79	-	-
	Single-branch (Fused)	**0.34**	**2.31×**	-
Overall System	TSA-Net (Unfused)	5.01	-	∼199
	TSA-Net (Fused)	**4.35**	**1.15×**	**∼230**

**Table 5:** Ablation results of our model and its variants.

Method	F1-Score		
	SMD	SMAP	MSL
TSA-NET	0.9782	0.9297	0.9389
w/o Local-STF	0.9660	0.8714	0.8896
w/o Global-STF	0.9679	0.8735	0.8957
w/o Feedback	0.9731	0.9072	0.8458
w/o Gated Fusion	0.9679	0.8676	0.8416
w/o Transformer	0.9582	0.8869	0.7806
w/o RepVGG	0.9655	0.8812	0.8530

**Table 6:** Ablation study on the number of attention heads (*h*) using the SMD dataset (D=38). The proposed strategy (h=19) effectively avoids feature subspace collapse while maximizing diversity.

Head Count (*h*)	Dim/Head ($d_{k}$)	Test MSE	Configuration Strategy
1	38	0.0030	Single-Head Baseline
2	19	0.0040	Conservative Partitioning
19	2	**0.0025**	Proposed (Dynamic Rule)
38	1	0.0031	Fully-Split (Limiting Case)

## Data Availability

Publicly available datasets were analyzed in this study. The SMAP and MSL datasets can be found at https://github.com/khundman/telemanom (accessed on 2 February 2026). The SMD dataset can be found at https://github.com/NetManAIOps/OmniAnomaly (accessed on 2 February 2026).
